# Expression of Concern: Downregulated brain and muscle aryl hydrocarbon receptor nuclear translocator-like protein-1 inhibits osteogenesis of BMSCs through p53 in type 2 diabetes mellitus

**DOI:** 10.1242/bio.055525

**Published:** 2020-10-05

**Authors:** Xiaofei Mao, Xiaoguang Li, Wei Hu, Siwei Hao, Yifang Yuan, Lian Guan, Bin Guo

The Editor-in-Chief of Biology Open has raised queries concerning the data shown in Biology Open (2020) 9, bio051482 (doi:10.1242/bio.051482)

The authors have been contacted for an explanation and have been asked to supply the original data.

Biology Open is publishing this note to make readers aware of the problem; we will provide further information once it has been resolved. This course of action follows the advice set out by COPE (Committee on Publication Ethics), of which Biology Open is a member.

